# An Insight into T-DNA Integration Events in *Medicago sativa*

**DOI:** 10.3390/ijms18091951

**Published:** 2017-09-12

**Authors:** Alessandro Nicolia, Nicoletta Ferradini, Fabio Veronesi, Daniele Rosellini

**Affiliations:** Dipartimento di Scienze Agrarie, Alimentari e Ambientali, Università degli Studi di Perugia, 06121 Perugia, Italy; nicoletta.ferradini@gmail.com (N.F.); fabio.veronesi@unipg.it (F.V.); daniele.rosellini@unipg.it (D.R.)

**Keywords:** Alfalfa, T-DNA integration, *Agrobacterium tumefaciens*, genetic engineering, vector backbone

## Abstract

The molecular mechanisms of transferred DNA (T-DNA) integration into the plant genome are still not completely understood. A large number of integration events have been analyzed in different species, shedding light on the molecular mechanisms involved, and on the frequent transfer of vector sequences outside the T-DNA borders, the so-called vector backbone (VB) sequences. In this work, we characterized 46 transgenic alfalfa (*Medicago sativa* L.) plants (events), generated in previous works, for the presence of VB tracts, and sequenced several T-DNA/genomic DNA (gDNA) junctions. We observed that about 29% of the transgenic events contained VB sequences, within the range reported in other species. Sequence analysis of the T-DNA/gDNA junctions evidenced larger deletions at LBs compared to RBs and insertions probably originated by different integration mechanisms. Overall, our findings in alfalfa are consistent with those in other plant species. This work extends the knowledge on the molecular events of T-DNA integration and can help to design better transformation protocols for alfalfa.

## 1. Introduction

*Agrobacterium tumefaciens* is the agent of the crown gall disease, determined by the transfer and permanent integration of bacterial oncogenes into the genome of infected plant cells. This process is a natural, cross-kingdom genetic transformation [1,2]. The molecular mechanisms at the basis of the crown gall disease are well elucidated and its molecular machinery has been exploited to transfer genes of interest into plants, opening the way to the development of plant genetic engineering.

Plant transformation mediated by *Agrobacterium tumefaciens* is a mature technique, and since the first reports in the early 80s, numerous plant species have been genetically engineered, initially dicots and subsequently also monocots. *Agrobacterium*-based gene delivery has been constantly improved. Initially, the natural tumor inducing (Ti) plasmid, containing both the virulence (*Vir*) genes and the transferred DNA (T-DNA), was disarmed by substituting the oncogenes in the T-DNA with the genes of interest. Subsequently, the limitations associated with the large size of the Ti vector were circumvented by the adoption of the binary vector system, in which the disarmed Ti plasmid still holds the *Vir* genes (helper vector) whereas the T-DNA is harbored by a second shuttle vector, assembled using *Escherichia coli* (binary vector).

The binary vector system is based on the fact that the *Vir* genes can act in *trans*, allowing the excision of the single strand T-DNA (ssT-DNA) from any other vector, present into the bacterium, in which the T-DNA is delimited by two 25 bp imperfect repeat sequences named Left and Right Border (LB, RB). The LB and RB sequences are very similar, therefore recognition as initiation or termination sites of ssT-DNA synthesis depends on surrounding sequences [3].

While the molecular mechanisms of ssT-DNA synthesis and transfer to plant cells are well known, those of integration into the plant genome are still not completely understood. A large number of integration events have been analyzed in different species with the objective of deducing the molecular events involved (Table 1). Moreover, by using model species (e.g., yeast, Arabidopsis, tobacco, maize) it has been possible to identify some of the molecules involved in the integration process [4,5]. The main findings can be summarized as follows:

1. Upon activation of the *Vir* gene cascade the ssT-DNA is released by the action of VirD1/VirD2 endonuclease complex; such mechanism involves the transfer of the sequence between the RB (5′-end) and LB (3′-end) to the host cells through a type IV secretion system [6,7].
2. Sequences exceeding the borders and belonging to the so-called vector backbone (VB) are frequently transferred along with the ssT-DNA [3]; other bacterial DNA may also enter the plant cell, including plasmid DNA [8,9] and chromosomal DNA [10,11,12].
3. Coordinated activity of bacterial and host proteins are necessary for infection and T-DNA integration, the latter largely relying on host factors [13,14].
4. T-DNA integration into the plant genome follows an “illegitimate” model, that is, integration is random and not directed by sequence identity, but rather by sequence micro similarities between the borders and the genome [15,16].
5. The ssT-DNA molecules can be directly integrated according to the model of Tinland [17]; however, such model does not explain the production of complex T-DNA insertions. Therefore, a model whereby the ssT-DNA is converted into double stranded T-DNA (dsT-DNA) prior to integration was proposed [5,18,19,20].
6. The presence of the so-called filler DNA, that is, DNA sequences from unknown sources often found between tandemly repeated copies of T-DNA or between the borders and the gDNA, points to a role of the double strand break (DSB) repair machinery in T-DNA integration [5]. The dsT-DNA, that seems to be abundant in Agrobacterium infected plant cells [16], can be recruited by the plant cell’s own DSB repair machinery, thus leading to end joining between ds-molecules and/or integration; this pathway could represent the most likely route for T-DNA integration [21]. Notably, Singer et al. [16] observed the formation of complex T-DNA circular structures in infected cells resembling the observed complex patterns of integration. Recently, van Kregten et al. [22] demonstrated the involvement of polymerase theta (Pol θ), a DSB repair enzyme, in T-DNA integration in Arabidopsis: the primer–template switching ability of this polymerase can explain the presence of filler DNA.
7. The availability of DSBs could be a limiting factor in the integration of T-DNA; however, a role of other types of lesions, such as single strand breaks (SSBs) cannot be excluded [4]. DSBs can be repaired either by the non-homologous end joining (NHEJ) pathway or by the homologous recombination (HR) pathway; the former seems to be the most frequent in plants, although the results are contradictory and a potential influence of the method of transformation used and of the cell type and/or developmental stage have been suggested. The evidences on the chromatin modifications that are essential for the DSB repair response and important for T-DNA integration have been reviewed, and models of integrations proposed [4].

Genetic engineering has been successfully established in different legume species, including alfalfa (*Medicago sativa* L., 2*n* = 4*x* = 32) a very important forage crop worldwide, permitting to introduce several useful traits [23]. However, a molecular analysis of T-DNA integration events in alfalfa has not been reported. In this work we characterized several transgenic plants (events), generated in previous works [24,25,26], for the presence of VB tracts, and sequenced several T-DNA/genomic DNA junctions.

## 2. Results

### 2.1. Isolation of Sequences Flanking T-DNA Insertions

#### 2.1.1. LB Junctions

**Figure 1:**
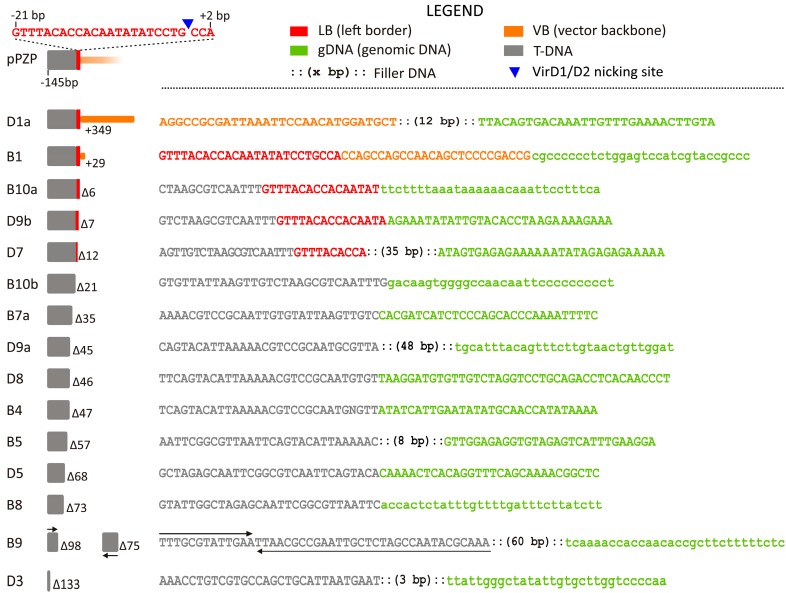
Analysis of left border (LB) junctions. Color codes are shown in the legend and the pPZP LB structure and sequence, in scale, is provided in the upper left corner. From left to right, each junction is described by: (1) an alphanumeric code identifying the transgenic event, if multiple junctions are isolated from the same event, these are identified by a lowercase letter; (2) a graphical representation, in scale, of the rearrangement occurred at the LB, along with the number of the deleted (Δ) or inserted (+) bp, in comparison with the expected intact transferred DNA (T-DNA) sequence (in one case T-DNA sequences with different orientations were detected, black arrows); (3) the sequence showing the 30 bp 5′ and 3′ of the junction; letters in lowercase indicate putative gDNA sequences (not verifiable by BLAST analysis, Table S1). Letters in red identify bases belonging to the LB.

The junctions between the LB and the gDNA were characterized in 13/46 events (28%). Two sequences were amplified from each of two transgenic events (B10 and D9), thus indicating the integration of two distinct T-DNAs (Figure 1). In two events (B1 and D1a) the junctions were between VB sequences, linked to an intact LB, and the gDNA (Figure 1): 26 and 346 bp of VB beyond the LB were transferred in these events, respectively. In one event (B9) the presence of a short inverted repeat of a T-DNA sequence was found.

Sequences characterized by an intact LB with the adjacent VB without any detectable junction with gDNA, were amplified in 7/46 events (B7b, B10c, B12, B13, D1b, D4, D6; not shown), indicating cases of large VB integrations (see below).

Precise junctions involving an intact LB were not found. Deletions of the T-DNA of variable sizes at the LB-gDNA junctions were identified, ranging from 6 bp (B10a) to 133 bp (D3) (Figure 1). Filler DNA was detected at the junction site in 6/13 events (46%) with sizes ranging from 3 (D3) to 60 bp (B9) (Figure 1). Filler DNA contained short (7–15 bp) patches of identity with vectors sequences (Figure S1).

#### 2.1.2. RB Junctions

**Figure 2:**
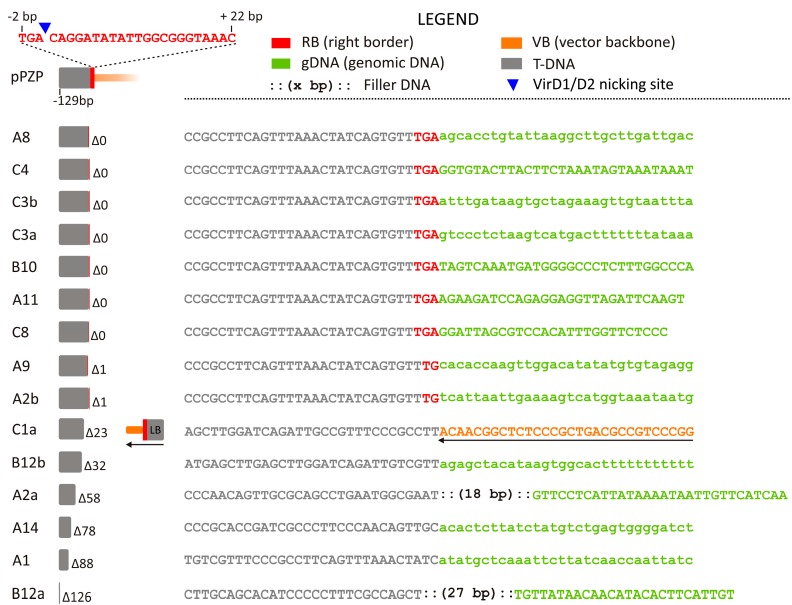
Analysis of the right border (RB) junctions. Color codes are shown in the legend and a pPZP RB model, in scale, is provided in the upper left corner. From left to right, each junction is described by: (1) an alphanumeric code identifying the transgenic event, if multiple junctions are isolated from the same event, these are identified by a lowercase letter; (2) a graphical representation, in scale, of the rearrangement occurred during integration at the RB, along with the number of the deleted (Δ) bp, in comparison with the expected intact T-DNA sequence; (3) the sequence showing the 30 bp 5′ and 3′ of the junction; letters in lowercase indicate putative gDNA sequences (not verifiable by BLAST analysis, Table S1) Letters in red identify bases belonging to the RB.

The RB junctions with the gDNA were characterized in 12/46 (26.0 %) events, with two sequences amplified from the same event in 3 cases (A2, B12, C3), thus indicating the integration of two distinct T-DNAs (Figure 2). Precise junctions showing the expected VirD1/VirD2 nicking site (A8, C4, C3b, C3a, B10, A11, C8) or 1 bp deletions (A9, A2b) represent the large majority of the cases (9/15 or 60%, Figure 2).

Significant deletions ranged in size from 23 to 126 bp. In two of these events (A2a, B12a), filler DNA (27 and 18 bp, respectively, Figure 2) with similarities to vector sequences was detected (Figure S1).

In one case, a junction between a partly deleted RB and a VB sequence linked to an intact LB in inverted orientation was found (C1a, Figure 2). Sequences characterized by an intact RB joined to the VB without any detectable junction with gDNA, were found in 3/46 events (C1b, C5, C6; not shown), indicating cases of large VB integrations (see below).

### 2.2. Polymerase Chain Reaction (PCR) Detection of Vector Backbone Sequences

**Figure 3:**
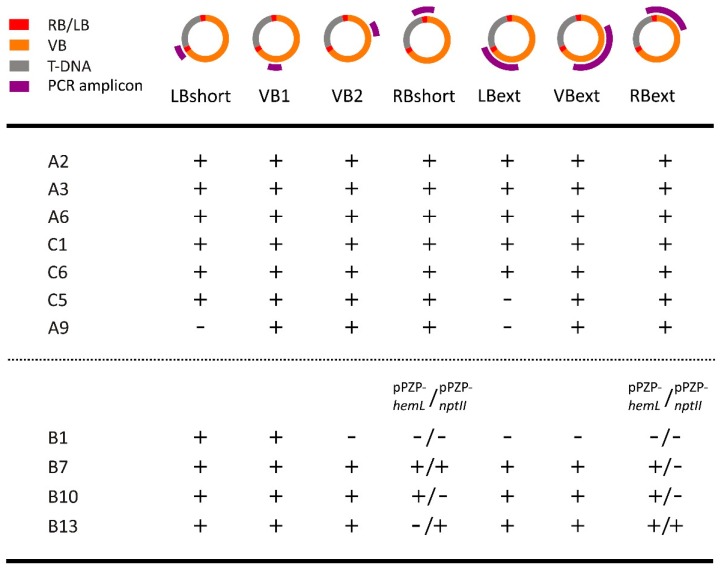
Summary of PCR screening results for the detection of vector backbone (VB) sequences in selected transgenic events of alfalfa. For the transgenic events produced by a co-transformation experiments (B1, B7, B10, B13) two columns are present for the amplicons RBshort and RBext to account for the two binary vectors used in the experiment (pPZP-*hemL*, pPZP-*nptII*, Figure S3). +, PCR positive; −, PCR negative.

In order to detect the transfer of VB sequences, a PCR screening was carried out using primers designed to cover the whole VB, with overlaps among amplicons (Figure 3 and Table S2). Sequences from the VB were detected in 29.7% (11/37) of the transgenic events: 26.6% in A plants, 30.4% in B plants, 33% in C plants (Table 2).

**Figure 4:**
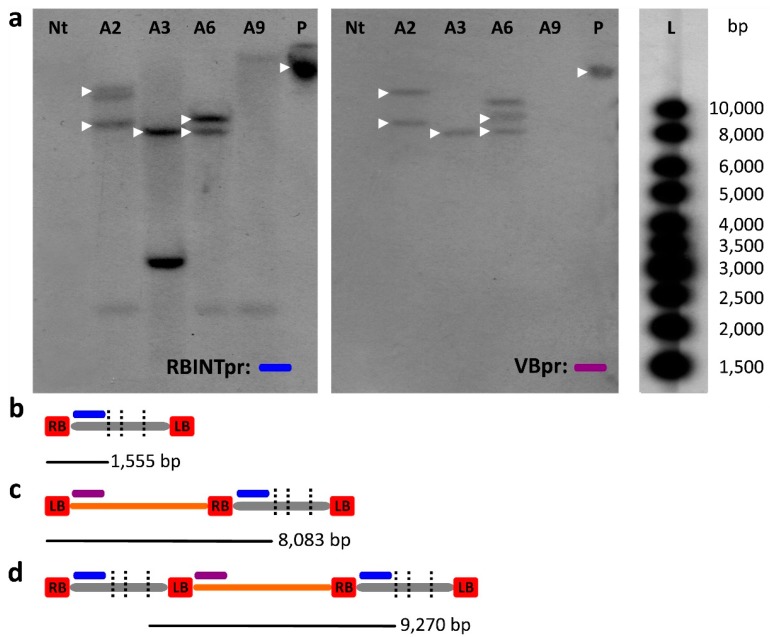
(**a**) Southern hybridization of genomic DNA extracted from T1 A plants with probe RBINTpr (blue segment) or VBpr (purple segment). The bands that hybridized to both probes are marked with a white triangle. Nt: non transgenic; P: binary vector pPZP-*hemL*-*nptII* (not linearized); L: 1 Kb ladder; (**b**–**d**) schemes (not in scale) of the restriction fragments produced by *Nco*I digestion (black vertical dotted lines are *Nco*I sites); (**b**) canonical T-DNA processing; (**c**) wrong initiation at the LB and transfer of whole VB along with a single copy of T-DNA; (**d**) correct initiation at the RB and incorrect termination at the LB, resulting in the transfer of the whole VB sequence along with two T-DNA copies. The position of the probes and the length of the restriction fragments are indicated.

Among A plants, three events (A2, A3, A6; Figure 3) were positive for all the primer combinations, suggesting that two T-DNAs were transferred along with the entire VB (Figure 4d). A9 was negative for both the primer combinations at the LB (Figure 3), so a model may be hypothesized where a VB sequence adjacent to the RB was transferred (Figure 4c).

PCR results on B Plants, deriving from a co-transformation experiment, are more difficult to interpret, due to the fact that some of the primer combinations (LBshort, VB1, VB2, LBext and VBext) amplify both vectors (Figure 3).

B1 was positive only for LBshort and VB1, but negative for LBext: this indicates two different integrations events, one of which has only a small residual fragment of VB left in the genome, probably due to a major rearrangement (Figure 3). Event B7 was negative for RBext (pPZP-*nptII*) whereas all the other amplicons, including RBshort (pPZP-*nptII*), were obtained; this gives an indication for a model where at least two T-DNA integration events contain VB sequences. Event B10 was negative for RBshort (pPZP-*nptII*) and RBext (pPZP-*nptII*): in this event apparently only one of the two vectors contributed to the transfer of VB sequences. B13 was negative for RBshort (pPZP-*hemL*), but positive for RBext (pPZP-*hemL*): this may be explained by an alteration of a primer binding site within the integrated VB sequence (Figure 3).

The results for C plants suggest the complete integration of the whole vector in C1 and C6 (positive for all the primer combinations). C5 was negative for LBext but positive for LBshort, suggesting that at least two T-DNA integration events contain VB sequences (Figure 3).

PCR amplification of the *A. tumefaciens picA* chromosomal gene was negative for all the 37 VB-positive transgenic events (Figure S2), demonstrating that the PCR results for VB integration were not affected by bacterial contamination of the DNA samples.

### 2.3. Southern Blot Analysis

**Figure 5:**
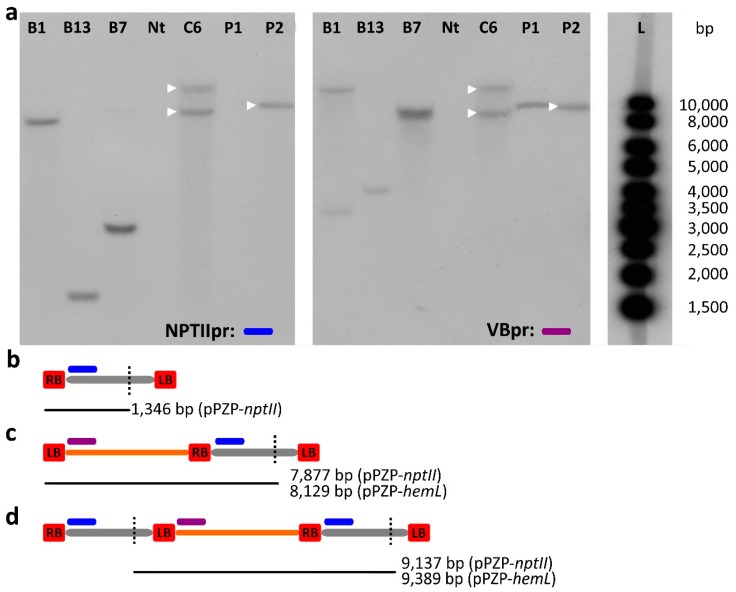
(**a**) Southern blot of genomic DNA extracted from T_1_ B and C plants with probe NTPIIpr (blue segment) and VBpr (purple segment). The bands that hybridized to both probes are marked with a white triangle. Nt: non transgenic; P1: binary vector pPZP-*hemL* (linearized); P2: binary vector pPZP-*nptII* (linearized); L: 1 Kb ladder; (**b**–**d**) schemes (not in scale) of the restriction fragments produced by *Nco*I (black vertical dotted line) digestion ; (**b**) a canonical T-DNA processing; (**c**) wrong initiation at the LB and transfer of the whole VB along with a single copy of the T-DNA; (**d**) correct initiation at the RB and an incorrect termination at the LB, resulting in the transfer of the whole VB sequence along with two T-DNA copies. The position of the probes and of the restriction sites, and the length of the restriction fragments are indicated.

To confirm PCR-based evidence of the transfer of VB sequences, we carried out a Southern blot analysis on the T1 progenies of selected events. Through the combination of a restriction enzyme not cutting the VB sequence (*NcoI*) and the design of two probes hybridizing to the T-DNA and to the VB, respectively (Figure 4 and Figure 5), we detected restriction fragments containing a T-DNA linked to the VB sequences.

In detail, a restriction fragment of 9270 bp for A plants, 9389 or 9137 bp for B plants (pPZP-*hemL* or pPZP-*nptII* respectively), and 9137 bp for C plants was expected in the case of the model depicted in Figure 4d and Figure 5d. In the cases of Figure 4c and Figure 5c, restriction fragments larger than 8083 bp for A plants, 7877 or 8129 bp for B plants (pPZP-*hemL* and pPZP-*nptII* respectively) and 7877 bp for C plants, were expected, depending on the position of the first *NcoI* site on the gDNA adjacent to the LB.

Considering the selected A plants (Figure 4), hybridization with the probe RBINTpr provided an estimation of the number of T-DNA loci in the plant genome, which was between 2 and 3 (Figure 4a). The presence of VB sequences linked to the T-DNA was verified by re-probing with the probe VBpr, that hybridized to some of the bands previously marked by the RBINTpr probe (Figure 4a).

The expected band of 9270 bp, consistent with the model depicted in Figure 4d, was detected only in one case (A6 in Figure 4a), whereas a band of about 8083 bp, in agreement with the model depicted in Figure 4c, was detected in two cases (A3 and A6, Figure 4a).

Interestingly, event A6, whose transgenic parent A6 was positive for all the VB amplicons (Figure 3), seemed to have inherited two distinct integration events characterized by the complete transfer of VB sequences, according to both the models depicted in Figure 4c,d.

Notably, event A2, which was positive for all the VB amplicons (Figure 3), showed two bands when re-probed with VBpr, with sizes larger than 8083 pb, compatible with the model depicted in Figure 4c.

The bands observed with probe RBINTpr and not with VBpr were attributed either to backbone-free T-DNA integration events or events containing VB sequences not detectable by the probe VBpr; a weak band between 2000 and 2500 bp was visible in three cases (lane A2, A6, and A9, Figure 4a) but its low intensity and identical size in three events indicates a non-specific hybridization.

An unexpected band of about 10 Kb containing the VB sequence was evidenced with VBpr in A6 (Figure 4a), indicating an insertion of VB sequences without T-DNA, which would imply a case of T-DNA initiation at the LB and termination at the RB, or a case of model Figure 4c with deletion of the T-DNA. A9 was negative with the probe VBpr, in agreement with the PCR results (Figure 3).

Considering B and C plants (Figure 5a) the hybridization with the probe NPTIIpr provided an estimation of the number of T-DNA loci in the plant genome; however, in the case of B plants, derived from an experiment of co-transformation, we visualized only the T-DNA from one of the two vectors used (Figure 5, P1 and P2 lanes). The observed number of restriction fragments was between 1 and 2 in the tested events.

VB sequences linked to the T-DNA was revealed by the VBpr probe in events B1, B13, B7, and C6 (Figure 5a), confirming PCR results. C6 showed two restriction fragments, the shortest of which compatible with the model depicted in Figure 5d; this agrees with PCR results for C6 (Figure 3). The larger band (>8083) fits the model depicted in Figure 5c.

Interestingly, in one case (B7, Figure 5a) a restriction fragment compatible with the model depicted in Figure 5d was detected after hybridization with VBpr, but not by hybridization with NPTIIpr. Likely, in this event from co-transformation, only the *hemL*-containing T-DNA is linked to VB sequence, but not the *nptII*-containing T-DNA, as supported by the PCR results (Figure 3). The same hypothesis can explain the results observed for events B1 and B13 (Figure 5), that showed different bands in the two hybridizations.

## 3. Discussion

In the alfalfa transgenic events analyzed in this work, we observed an average frequency of VB integration of 29.7% (Table 2). This percentage is considerably lower compared to what previously reported in the literature for *M. truncatula* (56%) [30] and other species (up to 90% in strawberry) [44] (Table 1).

Interestingly, Oltmanns et al. [27] in an experiment of genetic transformation of Arabidopsis and maize used different origins of replications for the binary vector and different strains of *Agrobacterium*, showing that the frequency of VB integration can be influenced by multiple factors: plant species, binary vector, strain, transformation method, target tissue. Other works, where single factors where kept constant, support this evidence [31,47,48]. As a consequence, the different experiments reported in the literature are difficult to compare. For instance, with the strain LBA4404, one of the two strains used in this work, VB integration frequencies between 0% and 90% have been reported (Table 1).

Oltmanns et al. [27] observed that launching the T-DNA from the *Agrobacterium* chromosome strongly reduced the frequency of integration of sequences exceeding the LB and RB. In fact, in the case of incorrect termination at LB, long ssT-DNA are released (theoretically as long as the entire *Agrobacterium* chromosome) and although the transfer of very long single strand sequences is possible, it is less frequent than the transfer of the relatively short T-DNA. In other words, a negative correlation between the size of the T-DNA and the frequency of VB integration exists [27]. Indeed, in our work the longest T-DNA showed the lowest frequency of VB integration (26.6%, Table 2 and Figure S3); however, this hypothesis was not statistically testable.

The current model of VB inclusion in the transferred T-DNA mainly relies on the different nature of the sequences surrounding the LB and RB. In particular, the LB can be recognized either as initiation or termination signal during T-strand production generating the two types of insert structures depicted in Figure 4c,d. On the contrary, the RB is not efficient as termination signal, because it is surrounded by the so called “overdrive” sequence, that strongly promote the initiation process [3,42].

In this work, the pPZP201BK-derived binary vectors have nopaline derived borders, with overdrive at RB [49] and both types of T-DNA structure inclusive of VB sequences (see above) were expected, as demonstrated in some transgenic events by Southern hybridization analysis.

Interestingly, Wenck et al. [9] hypothesized that an unbalanced ratio of the *Vir* genes versus the number of borders may result in an inefficient nicking by the VirD1/VirD2 complex, thus increasing the chance of VB integration. The binary vectors used in this work features a pVS1 origin, that ensures from 7 to 10 copies per *Agrobacterium* cell [27]. In the binary systems the helper plasmid is usually present in one or two copies per cell, so in our experimental conditions we likely had a *Vir*/border ratio between 2:7 and 1:10.

Particularly, Vain et al. [50] showed that the single addition of a *virG* gene, whose function is to act as transcriptional activator of the entire Vir pathway, abolished VB integration in rice.

In this work, we were able to isolate at least one sequence flanking the insertion site from about 52% of the events analyzed (Table 2), a success rate in line with that reported for the TAIL-PCR procedure [51].

The analysis of the flanking sequences revealed that the RB is less affected by rearrangements compared to the LB, in agreement with previous observations in other species [32,35,42,52,53,54]. All the intact LBs were associated with adjacent VB sequences (Figure 1), whereas intact RBs, showing precise junction with gDNA, were isolated in about half of the cases (Figure 2).

Insertions characterized by intact or partially deleted borders without filler DNA and not showing complex T-DNA structure (e.g., tandem repeats) can be explained by the model based on the integration of ssT-DNA [17]. In short, the LB (3′ end) first anneal to a short stretch of complementary gDNA and is subsequently trimmed, originating the frequently observed deletions at LB; in a second step the RB (5′ end), that is still bound to the VirD2 protein, anneals to the gDNA and VirD2 may assists ligation before being released [5].

We observed the presence of filler DNA up to 60 bp in a few integration events, more frequently associated with the LB than with the RB; in both cases it was never detected along with intact borders (Figure 1 and Figure 2). The filler DNA showed patch similarity with vector sequences (Figure S1). The presence of filler DNA can be associated with the DSB repair (DSBR) model of integration. According to Tzfira et al. [5], the ssT-DNA is first converted into dsT-DNA and, in proximity of a DSB in the gDNA, the four double strand ends can be processed by exonucleases, so that the single strand stretches can anneal in areas of microsimilarity and ligate. During synthesis-dependent repair, template switch can occur, which explains the presence of filler DNA. These mechanisms may explain the complex insertions characterized at LB (B9, Figure 1) and at RB (C1a, Figure 2).

Recently, the involvement of polymerase theta in T-DNA integration was unequivocally demonstrated in Arabidopsis, showing that the DSB repair mechanism is the main route to T-DNA integration; the model proposed explains the nature of filler DNA and does not involve the synthesis of dsT-DNA [22]. However, other mechanism can also play a role, and differences can exist among plant species.

## 4. Materials and Methods

### 4.1. Plant Materials

Forty six transgenic plants (events) were analysed in this work (Table 2); they were obtained from different transformation experiments using the alfalfa genotype RSY1 selected from the RegenS-Y germplasm [55]; the binary vectors were based on pPZP201BK [49], and the *A. tumefaciens* strains were either LBA4404 (plants A–C) or AGL1 (plants D) [24,25,26]. According to the vectors used in the transformation experiments, the plants were divided into four groups: A, transformed with the pPZP-*nptII*-*hemL* vector (15 events); B, co-transformed with the pPZP-*nptII* and pPZP-*hemL* vectors (13 events); C, transformed with the pPZP-*nptII* vector (9 events); and D, transformed with the pPZP-*MsGSAgr* vector (9 events).

### 4.2. Isolation of Sequences Flanking T-DNA Insertions

Total gDNA was extracted from young, fully expanded leaves collected from the 46 transgenic lines (events), using the GeneElute Plant Genomic DNA Miniprep Kit (SIGMA, St. Louis, MO, USA). Amplification of the T-DNA flanking sequences was carried out on the 46 gDNAs by hi-TAIL PCR according to the protocol of Liu and Chen [51]. Primer for this work were purchased from SIGMA and their sequences are reported in Table S3. Two combinations of Longer Arbitrary Degenerated (LAD) primers were tested (LAD1 + LAD3, LAD3 + LAD4) [51] to increase the chance of successful amplification of the T-DNA flanking regions and to find the optimal combination with three nested T-DNA-specific primers. Three LB nested primers (LBn1, LBn2, LBn3) were designed for B, C and D plants at position -669, -374 and -170 respectively, assuming as base zero the base immediately 5′of the VirD2 nicking site (Figure 1); similarly, three LB nested primers were designed for A plants (LBnA1, LBnA2, LBnA3) at position -276 ,-170 and -60, respectively (Figure S3).

Three RB nested primers (RBn1, RBn2, RBn3), were designed for B, C and D plants at positions -469, -356 and -154, respectively (Figure 2 and Figure S3); for A plants the primers LBn1 (-637), LBn2 (-343) and RBn3 (-155) were used to amplify at RB.

For each transgenic line, the second and third nested hi-TAIL PCR reaction were subjected to electrophoresis in 1.5% agarose gels. The third nested reactions were purified (Wizard SV Gel and PCR Clean-up System, Promega, Madison, WI, USA) when the expected shift of amplicon sizes from the nested PCRs was observed; the amplicon was cloned in the pGEM-T vector (pGEM-T Vector Systems, Promega, Madison, WI, USA) and double strand sequenced (Macrogen, available online: www.macrogen.com). The software AlignX (Thermo Scientific, Waltham, MA, USA) was used to identify the junction between the LBs or RBs and the plant genome. These flanking sequences were subsequently used to validate the gDNA sequences by searching the NCBI databases (Available online: https://blast.ncbi.nlm.nih.gov/Blast.cgi); only BLAST results having a similarity equal or greater than 70% with the query were considered for validation (Table S1).

### 4.3. PCR Detection of Vector Backbone Sequences

Specific primer pairs covering the VB (Figure 3 and Figure S3) were designed using the software Primer3 [56]. Only transgenic events belonging to group A, B and C were included in this analysis. The primer combinations and the thermal cycling conditions are shown in Table S2; the expected amplicons are graphically described in Figure 3. PCR reactions were carried out in 50 µL using 1× Buffer, 1.5 mM MgCl2, 0.2 mM dNTPs, 0.4 µM primers, 1U Taq (SIGMA) and 30 ng genomic DNA. For difficult amplicons, PCR reactions were carried out with Phusion polymerase (Thermo Scientific) in 50 µL using 1× Buffer GC, 3% DMSO, 0.2 mM dNTPs, 0.5 µM primers, 1 U of polymerase and 100 ng genomic DNA.

To check for any *Agrobacterium* contamination of the gDNA samples a PCR was carried out with the primers PICAFOR and PICAREV (Table S3), specifically designed to amplify a 432 bp fragment within the *picA* locus of the *Agrobacterium* chromosome [57]. Thermal cycling conditions were 94 °C for 10 min, 40 cycles at 94 °C for 30 s, 66 °C for 30 s and 72 °C for 30 s.

PCR reactions were subjected to electrophoresis in 1.2% agarose gel.

### 4.4. Southern Hybridization Analysis

Eight transgenic events (A2, A3, A6, A9, B1, B7, B13, C6) were selected on the basis of the PCR screening for VB sequences and crossed with the unrelated genotype “Classe” used as pollen donor. Total genomic DNA was extracted from 20 seedlings for each cross and screened by PCR for the presence of the transgenes as reported [24,26].

One PCR-positive T1 plant per cross was selected and 30 µg of genomic DNA used for Southern hybridization analysis following standard procedures [58]. Genomic DNA was digested overnight with NcoI-HF (NEB), resolved by electrophoresis in a 0.7% agarose gel overnight at 40 volts, depurinated by incubation in 0.25 M HCl, blotted by capillarity onto a nylon membrane (Hybond-N+, GE Healthcare, Chicago, IL, USA) and crosslinked at 80 °C for 2 h.

Two membranes were obtained, one containing the T1 samples A2, A3, A6, and A9, and one containing the T1 samples B1, B7, B13 and C6. The probes RBINTpr (319 bp), NPTIIpr (312 bp) and VBpr (257 bp) were generated by PCR using the primers RBINT For/Rev, NPTIIsh For/Rev and VB1 For/Rev, respectively (Table S3) using standard procedures [58]; their position is indicated in Figure 4 and Figure 5.

The membrane carrying group A samples was hybridized overnight with dCTP, α-32P (3000 Ci mmol^−1^, PerkinElmer, Waltham, MA, USA) radiolabelled probe RBINTpr, whereas the membrane with B and C samples was hybridized with the probe NPTIIpr. The membranes were then exposed 7 days at −80 °C to a Kodak Biomax ML film (Kodak, Rochester, NY, USA). The membranes were subsequently stripped in a boiling solution of 0.1% SDS and both re-hybridized with the radiolabeled probe VBpr. The lane of the two membranes containing the Gene Ruler 1 Kb DNA ladder (Thermo Scientific) was cut and hybridized separately.

## 5. Conclusions

The T-DNA integration research has shifted from analyzing flanking sequences to identifying the molecules involved in the integration process [5] that are, at large, those belonging to the DNA repair pathway. Although eukaryotes share common DNA repair mechanisms and significant progress was made in model organism (yeast, mammals and plants), there are species-specific differences that require to enlarge the number of organisms studied [59,60,61,62].

In alfalfa, no information was available on the patterns of DNA integration and VB transfer in *Agrobacterium*-mediated transformation, possibly because the lack of a genome sequence for this species has hindered these investigations. However, important biotechnological tools have been developed in the closely related, diploid model species *M. truncatula* (2*n* = 2*x* = 16), for which a genome sequencing project was completed [63] and a program of insertional mutagenesis was carried out [64,65].

In this work, we have characterized a number of transgenic alfalfa events, previously produced in our lab [24,25,26]. By sequencing insertion sites we showed that, as previously reported in other species [42], multiple mechanisms are probably involved in T-DNA integration in alfalfa. We also demonstrated the transfer and integration of VB sequences through *Agrobacterium* genetic transformation and their sexual transmission to progenies.

The quality of the insertions has received large attention worldwide by the scientific community and the regulatory bodies, with the aim of improving precision and minimizing the possible risks of the genetic modification of crop plants [66].

Possible ways to improve the quality of insertions in the alfalfa genome can be envisaged:

(a) Launching the T-DNA from the *Agrobacterium* chromosome may reduce the risk of transferring sequences belonging to the binary vector (including antibiotic resistance genes for bacterial selection); however, engineering the bacterial chromosome adds complication to the procedures, and a decrease in the transformation efficiency may result [27]; it should also be considered that rare cases of transfer of sequences belonging to *Agrobacterium* chromosome are documented [10,11,12];
(b) Increasing the number of LBs and *VirG* gene or including negative selectable marker genes in the VB would improve the correct processing of the borders and allow counter selection of events that include VB sequences [3,30,36,50,67];
(c) Using plant-derived sequences for vector construction (e.g., plant-derived SMGs, cisgenesis) [68,69,70] can relieve the perceived risk of genetically modified plants. The application of new breeding techniques such as genome editing is also offering new tools for precise modification of the alfafa genome.

## Figures and Tables

**Table 1:** Percentages of transgenic events containing vector backbone (VB) sequences in different plant species.

Species	Agrobacterium Strain	Vector	VB % *	References
Arabidopsis	EHA101, GV3101, LBA4404	pTF101.1, pTF::Bin19, pTF::UCD2, pTF:ri, pSDM1550, pITC15, pMAW2035HYG	0–68	[9,27,28]
Barley	AGL0	pVec8-GFP	48	[29]
Barrel medic	EHA105	pSIM843	56	[30]
Canola	ABI	pMON67438	15	[31]
Creeping bentgrass	EHA101	pPMI-GFP, pUHVA1, pAHVA1	3	[32]
Corn	ABI	pMON92726, pMON65153	30–33	[31,33]
Cotton	AGL1	pPZP-GFP	31	[34]
Grapevine	LBA4404	pGA643, pBH710	29–50	[35,36]
Maize	EHA101, GV3101, LBA4404	pTF101.1, pTF::Bin19, pTF::UCD2, pTF:ri	18–55	[27]
Petunia	LBA4404	pFLG5972	22	[37]
Potato	LBA4404	pSIM108	72	[38]
Rice	LBA4404, AGL1, EHA105	pCXa21K, pC30063, pGreen/pSOUP, pSK100/200, pEU334NA/NB, pNU393B2, pGA2144	4–60	[39,40,41,42]
Sorghum	LBA4404, AGL1	PHP32269	4–26	[43]
Soybean	ABI	pMON83326	40	[31]
Strawberry	LBA4404	pBINPLUS, pGUSINT	67–90	[44]
Tobacco	LBA4404, GV3101, EHA105	pBSG-1/BSG-2, pBH710	75–80	[8,36]
Tomato	LBA4404	pBH710	67	[36]
Wheat	AGL1	pCG181-1G+pCS167-1B, pCG185-1G+pCS167-1B, pCG185-2G+pAL154, pCG185-3G+pAL154, pCG185-4G+pAL154	8–62	[45,46]

* For each species the lowest and highest value reported in literature are indicated.

**Table 2:** Transgenic alfalfa events used for isolating flanking sequences (FS) and assessed for the presence of vector backbone (VB) sequences.

Plant Group	No. of Events	*Agrobacterium* Strain ^a^	Binary Vector	FS ^b^	VB
A	15	LBA4404	pPZP-*nptII-hemL*	6 (40.0%)	4 (26.6%)
B	13	LBA4404	pPZP-*hemL* + pZPZ-*nptII*	8 (61.5%)	4 (30.4%)
C	9	LBA4404	pPZP-*nptII*	4 (44.4%)	3 (33.3%)
D	9	AGL1	pPZP-*MsGSAgr*	6 (66.6%)	nt
Total	46			24/46 (52.2%)	11/37 (29.7%)

^a^ *Agrobacterium* strain used for transformation; ^b^ percentage of events (no. of positive/total plants tested) in which at least one T-DNA flanking sequence was isolated; nt, not tested.

## Supplementary Materials

Supplementary materials can be found at www.mdpi.com/1422-0067/18/9/1951/s1.

## References

[1] Kyndt T., Quispe D., Zhai H., Jarret R., Ghislain M., Liu Q., Gheysen G., Kreuze J.F. (2015). The genome of cultivated sweet potato contains *Agrobacterium* T-DNAs with expressed genes: An example of a naturally transgenic food crop. Proc. Natl. Acad. Sci. USA.

[2] Suzuki K., Yamashita I., Tanaka N. (2002). Tobacco plants were transformed by *Agrobacterium rhizogenes* infection during their evolution. Plant J..

[3] Podevin N., de Buck S., de Wilde C., Depicker A. (2006). Insights into recognition of the T-DNA border repeats as termination sites for T-strand synthesis by *Agrobacterium tumefaciens*. Transgenic Res..

[4] Magori S., Citovsky V. (2011). Epigenetic control of *Agrobacterium* T-DNA integration. Biochim. Biophys. Acta.

[5] Tzfira T., Li J., Lacroix B., Citovsky V. (2004). *Agrobacterium* T-DNA integration: Molecules and models. Trends Genet..

[6] Pitzschke A., Hirt H. (2010). New insights into an old story: *Agrobacterium*-induced tumour formation in plants by plant transformation. EMBO J..

[7] Tzfira T., Citovsky V. (2006). *Agrobacterium*-mediated genetic transformation of plants: Biology and biotechnology. Curr. Opin. Biotechnol..

[8] Kononov M.E., Bassuner B., Gelvin S.B. (1997). Integration of T-DNA binary vector “backbone” sequences into the tobacco genome: Evidence for multiple complex patterns of integration. Plant J..

[9] Wenck A., Czakó M., Kanevski I., Márton L. (1997). Frequent collinear long transfer of DNA inclusive of the whole binary vector during *Agrobacterium*-mediated transformation. Plant Mol. Biol..

[10] Ulker B., Li Y., Rosso M.G., Logemann E., Somssich I.E., Weisshaar B. (2008). T-DNA-mediated transfer of *Agrobacterium tumefaciens* chromosomal DNA into plants. Nat. Biotechnol..

[11] Kim S.-R., An G. (2012). Bacterial transposons are co-transferred with T-DNA to rice chromosomes during *Agrobacterium*-mediated transformation. Mol. Cells.

[12] Philips J.G., Naim F., Lorenc M.T., Dudley K.J., Hellens R.P., Waterhouse P.M. (2017). The widely used *Nicotiana benthamiana* 16c line has an unusual T-DNA integration pattern including a transposon sequence. PLoS ONE.

[13] Wang L., Lacroix B., Guo J., Citovsky V. (2017). The *Agrobacterium* VirE2 effector interacts with multiple members of the *Arabidopsis* VIP1 protein family. Mol. Plant Pathol..

[14] Li X., Pan S.Q. (2017). *Agrobacterium* delivers VirE2 protein into host cells via clathrin-mediated endocytosis. Sci. Adv..

[15] Gheysen G., Villarroel R., van Montagu M. (1991). Illegitimate recombination in plants: A model for T-DNA integration. Genes Dev..

[16] Singer K., Shiboleth Y.M., Li J., Tzfira T. (2012). Formation of complex extrachromosomal T-DNA structures in *Agrobacterium tumefaciens*-infected plants. Plant Physiol..

[17] Tinland B. (1996). The integration of T-DNA into plant genomes. Trends Plant Sci..

[18] De Buck S., Jacobs A., van Montagu M., Depicker A. (1999). The DNA sequences of T-DNA junctions suggest that complex T-DNA loci are formed by a recombination process resembling T-DNA integration. Plant J..

[19] De Neve M., de Buck S., Jacobs A., van Montagu M., Depicker A. (1997). T-DNA integration patterns in co-transformed plant cells suggest that T-DNA repeats originate from co-integration of separate T-DNAs. Plant J..

[20] Mayerhofer R., Koncz-Kalman  Z., Nawrath C., Bakkeren G., Crameri A., Angelis K., Redei G.P., Schell J., Hohn B., Koncz C. (1991). T-DNA integration: A mode of illegitimate recombination in plants. EMBO J..

[21] Hu Y., Chen Z., Zhuang C., Huang J. (2017). Cascade of chromosomal rearrangements caused by a heterogeneous T-DNA integration supports the double-stranded break repair model for T-DNA integration. Plant J..

[22] Van Kregten M., de Pater S., Romeijn R., van Schendel R., Hooykaas P.J., Tijsterman M. (2016). T-DNA integration in plants results from Polymerase-θ-mediated DNA repair. Nat. Plants.

[23] Kölliker R., Rosellini D., Wang Z.Y. (2010). Development and application of biotechnological and molecular genetic tools. Fodder Crops and Amenity Grasses.

[24] Ferradini N., Nicolia A., Capomaccio S., Veronesi F., Rosellini D. (2011). Assessment of simple marker-free genetic transformation techniques in alfalfa. Plant Cell Rep..

[25] Ferradini N., Nicolia A., Capomaccio S., Veronesi F., Rosellini D. (2011). A point mutation in the *Medicago sativa* GSA gene provides a novel, efficient, selectable marker for plant genetic engineering. J. Biotechnol..

[26] Rosellini D., Capomaccio S., Ferradini N., Savo Sardaro M.L., Nicolia A., Veronesi F. (2007). Non-antibiotic, efficient selection for alfalfa genetic engineering. Plant Cell Rep..

[27] Oltmanns H., Frame B., Lee L.-Y., Johnson S., Li B., Wang K., Gelvin S.B. (2010). Generation of backbone-free, low transgene copy plants by launching T-DNA from the *Agrobacterium* chromosome. Plant Physiol..

[28] Van der Graaff E., den Dulk-Ras A., Hooykaas P.J. (1996). Deviating T-DNA transfer from *Agrobacterium tumefaciens* to plants. Plant Mol. Biol..

[29] Lange M., Vincze E., Møller M.G., Holm P.B. (2006). Molecular analysis of transgene and vector backbone integration into the barley genome following *Agrobacterium*-mediated transformation. Plant Cell Rep..

[30] Confalonieri M., Borghetti R., Macovei A., Testoni C., Carbonera D., Fevereiro M.P., Rommens C., Swords K., Piano E., Balestrazzi A. (2010). Backbone-free transformation of barrel medic (*Medicago truncatula*) with a Medicago-derived transfer DNA. Plant Cell Rep..

[31] Ye X., Williams E.J., Shen J., Johnson S., Lowe B., Radke S., Strickland S., Esser J.A., Petersen M.W., Gilbertson L.A. (2011). Enhanced production of single copy backbone-free transgenic plants in multiple crop species using binary vectors with a pRi replication origin in *Agrobacterium tumefaciens*. Transgenic Res..

[32] Fu D., St. Amand P.C., Xiao Y., Muthukrishnan S., Liang G.H. (2006). Characterization of T-DNA integration in creeping bentgrass. Plant Sci..

[33] Huang S., Gilbertson L.A., Adams T.H., Malloy K.P., Reisenbigler E.K., Birr D.H., Snyder M.W., Zhang Q., Luethy M.H. (2004). Generation of marker-free transgenic maize by regular two-border *Agrobacterium* transformation vectors. Transgenic Res..

[34] Zhang J., Cai L., Cheng J., Mao H., Fan X., Meng Z., Chan K.M., Zhang H., Qi J., Ji L. (2008). Transgene integration and organization in cotton (*Gossypium hirsutum* L.) genome. Transgenic Res..

[35] Gambino G., Chitarra W., Maghuly F., Laimer M., Boccacci P., Torello M.D., Gribaudo I. (2009). Characterization of T-DNA insertions in transgenic grapevines obtained by *Agrobacterium*-mediated transformation. Mol. Breed..

[36] Hanson B., Engler D., Moy Y., Newman B., Ralston E., Gutterson N. (1999). A simple method to enrich an *Agrobacterium*-transformed population for plants containing only T-DNA sequences. Plant J..

[37] Cluster P.D., O’Dell M., Metzlaff M., Flavell R.B. (1996). Details of T-DNA structural organization from a transgenic Petunia population exhibiting co-suppression. Plant Mol. Biol..

[38] Rommens C.M., Humara J.M., Ye J., Yan H., Richael C., Zhang L., Perry R., Swords K. (2004). Crop improvement through modification of the plant’s own genome. Plant Physiol..

[39] Yin Z., Wang G.-L. (2000). Evidence of multiple complex patterns of T-DNA integration into the rice genome. TAG Theor. Appl. Genet..

[40] Sallaud C., Meynard D., van Boxtel J., Gay C., Bès M., Brizard J. P., Larmande P., Ortega D., Raynal M., Portefaix M. (2003). Highly efficient production and characterization of T-DNA plants for rice ( Oryza sativa L.) functional genomics. Theor. Appl. Genet..

[41] Afolabi A., Worland B., Snape J. W., Vain P. (2004). A large-scale study of rice plants transformed with different T-DNAs provides new insights into locus composition and T-DNA linkage configurations. Theor. Appl. Genet..

[42] Zhu Q.-H., Ramm K., Eamens A.L., Dennis E.S., Upadhyaya N.M. (2006). Transgene structures suggest that multiple mechanisms are involved in T-DNA integration in plants. Plant Sci..

[43] Wu E., Lenderts B., Glassman K., Berezowska K.M., Christensen H., Asmus T., Zhen S., Chu U., Cho M.J., Zhao Z.Y. (2014). Optimized *Agrobacterium*-mediated sorghum transformation protocol and molecular data of transgenic sorghum plants. In Vitro Cell. Dev. Biol. Plant.

[44] Abdal-Aziz S.A., Pliego A.F., Quesada M.A., Mercado J.A. (2006). Evidence of frequent integration of non-T-DNA vector backbone sequences in transgenic strawberry plant. J. Biosci. Bioeng..

[45] Wang G.P., Yu X.D., Sun Y.W., Jones H.D., Xia L.Q. (2016). Generation of marker- and/or backbone-free transgenic wheat plants via *Agrobacterium*-mediated transformation. Front. Plant Sci..

[46] Wu H., Sparks C.A., Jones H.D. (2006). Characterisation of T-DNA loci and vector backbone sequences in transgenic wheat produced by *Agrobacterium*-mediated transformation. Mol. Breed..

[47] De Buck S., Podevin N., Nolf J., Jacobs A., Depicker A. (2009). The T-DNA integration pattern in Arabidopsis transformants is highly determined by the transformed target cell. Plant J..

[48] Petti C., Wendt T., Meade C., Mullins E. (2009). Evidence of genotype dependency within *Agrobacterium tumefaciens* in relation to the integration of vector backbone sequence in transgenic Phytophthora infestans-tolerant potato. J. Biosci. Bioeng..

[49] Hajdukiewicz P., Svab Z., Maliga P. (1994). The small, versatile pPZP family of *Agrobacterium* binary vectors for plant transformation. Plant Mol. Biol..

[50] Vain P., Harvey A., Worland B., Ross S., Snape J.W., Lonsdale D. (2004). The effect of additional virulence genes on transformation efficiency, transgene integration and expression in rice plants using the pGreen/pSoup dual binary vector system. Transgenic Res..

[51] Liu Y.G., Chen Y. (2007). High-efficiency thermal asymmetric interlaced PCR for amplification of unknown flanking sequences. Biotechniques.

[52] Kim S.-R., Lee J., Jun S.-H., Park S., Kang H.-G., Kwon S., An G. (2003). Transgene structures in T-DNA-inserted rice plants. Plant Mol. Biol..

[53] De Buck S., De Wilde C., Van Montagu M., Depicker A. (2000). T-DNA vector backbone sequences are frequently integrated into the genome of transgenic plants obtained by *Agrobacterium*-mediated transformation. Mol. Breed..

[54] Kumar S., Fladung M. (2002). Transgene integration in aspen: Structures of integration sites and mechanism of T-DNA integration. Plant J..

[55] Bingham G. (1991). Registration of alfalfa hybrid Regen-Sy germplasm for tissue culture and transformation research. Crop Sci..

[56] Rozen S., Skaletsky H. (1999). Primer3 on the WWW for general users and for biologist programmers. Bioinformatics Methods and Protocols.

[57] Rong L.J., Karcher S.J., Gelvin S.B. (1991). Genetic and molecular analyses of picA, a plant-inducible locus on the *Agrobacterium tumefaciens* chromosome. J. Bacteriol..

[58] Sambrook J., Russell D.W. (2012). Molecular Cloning.

[59] Kirik A., Salomon S., Puchta H. (2000). Species-specific double-strand break repair and genome evolution in plants. EMBO J..

[60] Manova V., Gruszka D. (2015). DNA damage and repair in plants-from models to crops. Front. Plant Sci..

[61] Orel N., Puchta H. (2003). Differences in the processing of DNA ends in *Arabidopsis thaliana* and tobacco: Possible implication for genome evolution. Plant Mol. Biol..

[62] Windels P., de Buck S., van Bockstaele E., de Loose M., Depicker A. (2003). T-DNA integration in *Arabidopsis* chromosomes. Presence and origin of filler DNA sequences. Plant Physiol..

[63] Tang H., Krishnakumar V., Bidwell S., Rosen B., Chan A., Zhou S., Gentzbittel L., Childs K. L., Yandell M., Gundlach H. (2014). An improved genome release (version Mt4.0) for the model legume Medicago truncatula. BMC Genom..

[64] Porceddu A., Panara F., Calderini O., Molinari L., Taviani P., Lanfaloni L., Scotti C., Carelli M., Scaramelli L., Bruschi G. (2008). An Italian functional genomic resource for Medicago truncatula. BMC Res. Notes.

[65] Scholte M., Erfurth I., Rippa S., Mondy S., Cosson V., Durand P., Breda C., Trinh H., Rodriguez -L.I., Kondorosi E. (2002). T-DNA tagging in the model legume Medicago truncatula allows efficient gene discovery. Mol. Breed..

[66] Nicolia A., Manzo A., Veronesi F., Rosellini D. (2014). An overview of the last 10 years of genetically engineered crop safety research. Crit. Rev. Biotechnol..

[67] Kondrák M., van der Meer I.M., Bánfalvi Z. (2006). Generation of marker- and backbone-free transgenic potatoes by site-specific recombination and a bi-functional marker gene in a non-regular one-border agrobacterium transformation vector. Transgenic Res..

[68] Rosellini D. (2011). Selectable marker genes from plants: Reliability and potential. In Vitro Cell. Dev. Biol. Plant.

[69] Rosellini D. (2012). Selectable markers and reporter genes: A well-furnished toolbox for plant science and genetic engineering. Crit. Rev. Plant Sci..

[70] Holme I.B., Wendt T., Holm P.B. (2013). Intragenesis and cisgenesis as alternatives to transgenic crop development. Plant Biotechnol. J..

