# Supplementary material for: An Insight into T-DNA Integration Events in Medicago sativa
Source: Int J Mol Sci. 2017 Sep 12;18(9):1951. doi: 10.3390/ijms18091951 (PMC5618600; doi:10.3390/ijms18091951)
Supplement: Supplementary file 1 [file ijms-18-01951-s001.pdf]

An insight into T-DNA integration events in *Medicago sativa*

Alessandro Nicolia, Nicoletta Ferradini, Fabio Veronesi and Daniele Rosellini

Supplementary materials

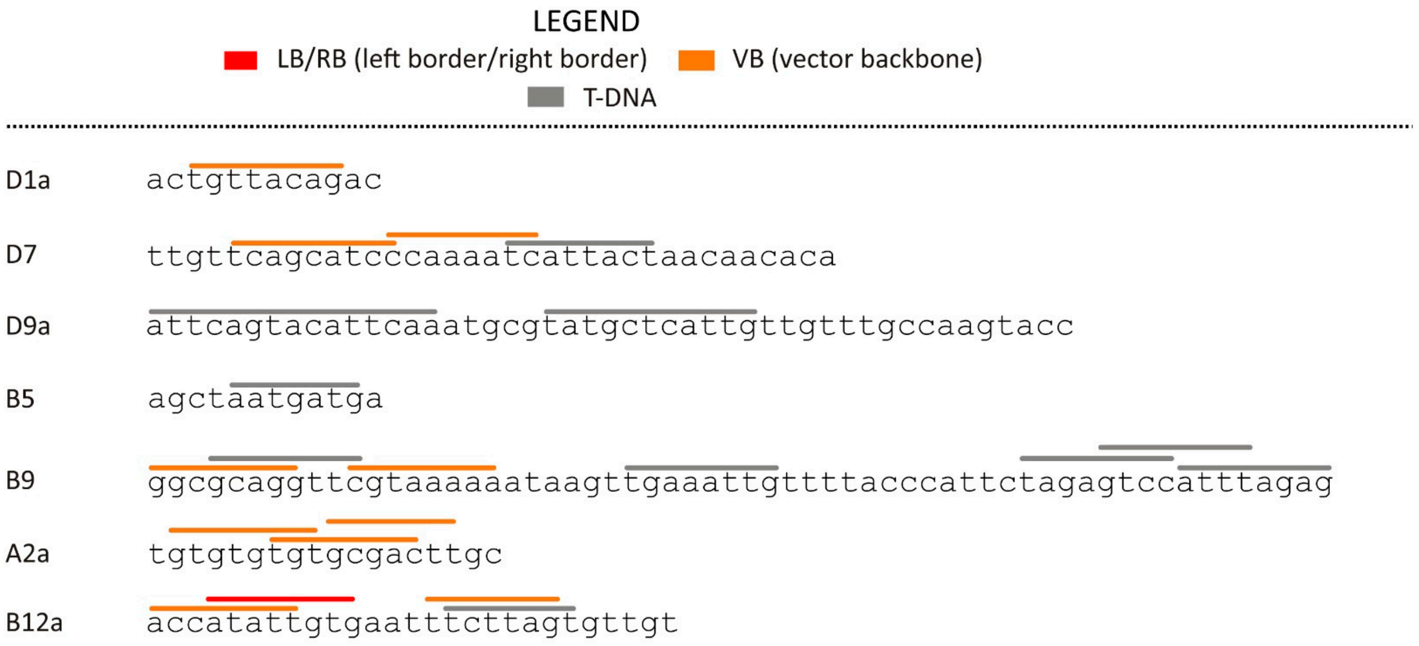

Figure S1. Results of the filler DNA alignment with sequences of the binary vectors. Nucleotide stretches showing 100% identity with vector sequences are marked by horizontal bars coloured accordingly to the legend.

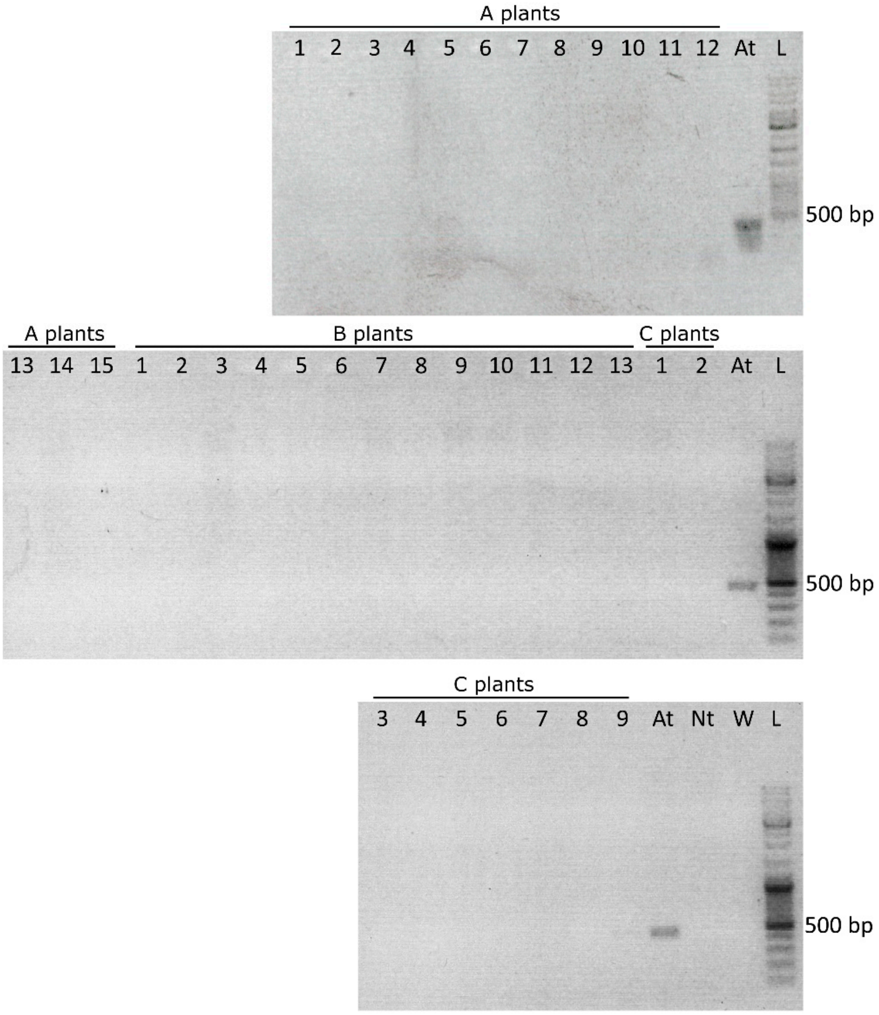

Figure S2. PCR screening to check for *Agrobacterium* contamination in the A, B and C plant genomic DNA (gDNA) samples. At: *Agrobacterium tumefaciens* gDNA; Nt: gDNA of a non transgenic plant; W: water; L: ladder 1Kb.

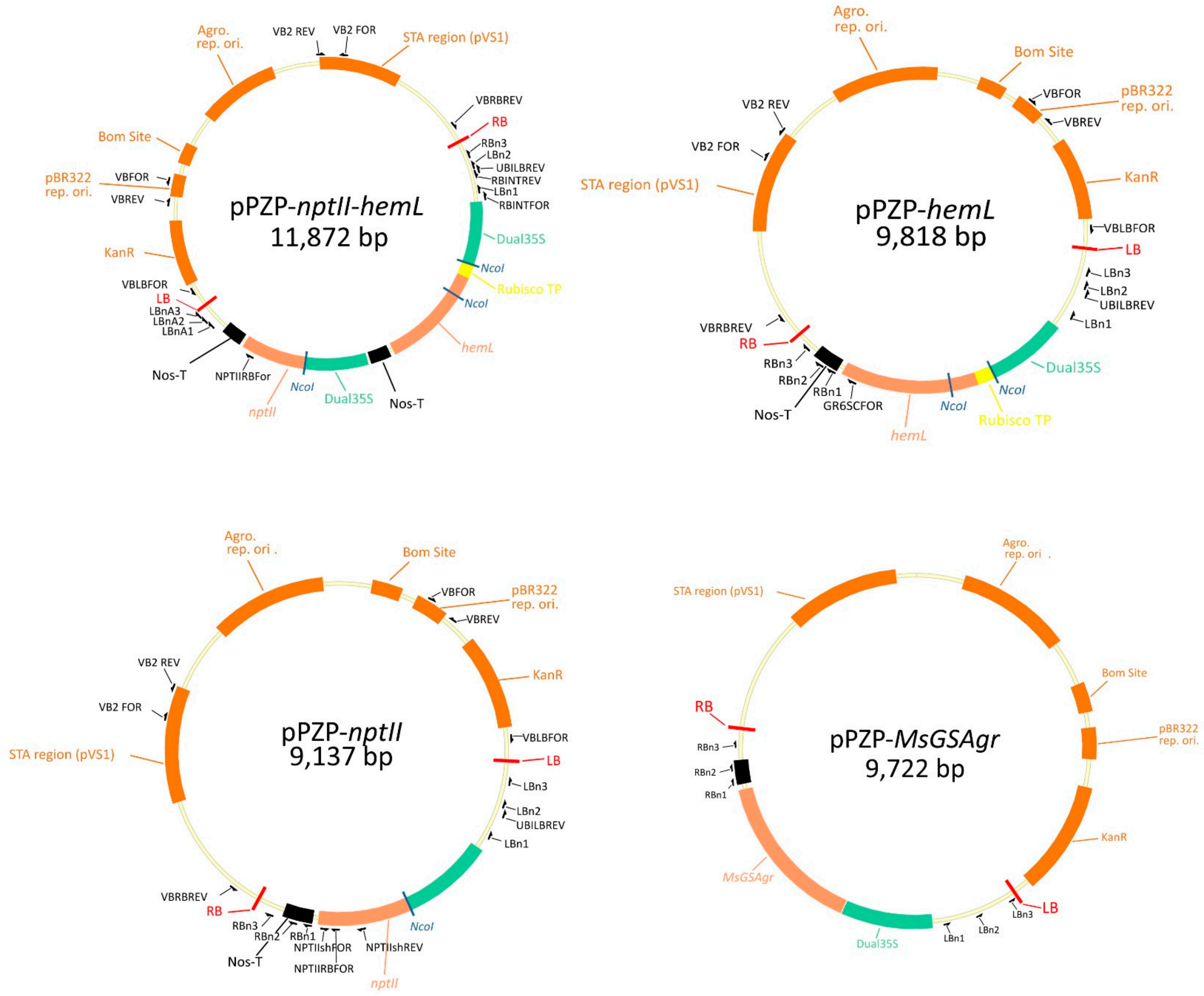

Figure S3. Maps of all vectors used to obtain the transgenic events analyzed in this work. The location of all primers is indicated.

Table S1. BLAST analysis of the gDNA sequences isolated from the T-DNA junctions

T-DNA junction	Best hit species	Gene Bank ID	Identity %[a]
Left border			
D1a	M. truncatula	AC144481	89.7 (269/300)
B1	nf	nf	nf
B10a	M. truncatula	AC134822	32.3 (52/161)
D9b	M. truncatula	CT030028	91.7 (188/205)
D7	M. truncatula	AC152498	84.4 (238/282)
B10b	nf	nf	nf
B7a	M. truncatula	AC151709	74.8 (232/310)
D9a	M. truncatula	AC239792	60.4 (81/134)
D8	M. truncatula	AC119409	88.4 (306/346)
B4	M. truncatula	AC146854	75.2 (490/652)
B5	M. truncatula	AC123899	72.3 (266/368)
D5	M. truncatula	AC124970	80.6 (987/1,225)
B8	M. truncatula	AC166287	30.4 (189/621)
B9	M. truncatula	CU302329	48.7 (139/285)
D3	M. truncatula	CR962125	67.1 (96/143)
Right border			
A8	L. japonicus	BT140217	16.7 (114/681)
C4	M. truncatula	CR940305	81.5 (440/540)
C3b	M. truncatula	FP102223	34.4 (162/471)
C3a	M. truncatula	CT025843	23.2 (72/310)
B10	M. truncatula	AC182815	77.7 (261/336)
A11	M. truncatula	CT573052	87.9 (210/239)
C8	G. max	XM_003522789	71.4 (407/570)
A9	V. vinifera	AM464998	23.8 (74/311)
A2b	nf	nf	nf
B12b	nf	nf	nf
A2a	M. truncatula	AC169181	84.6 (363/429)
A14	M. truncatula	AC149269	17.6 (49/278)
A1	M. truncatula	BT146992	15 (39/260)
B12a	M. truncatula	CT030234	82.4 (716/868)

a: percentage of identity obtained by the BLAST software (Query/Subject);
nf: not found.

Table S2 - PCR conditions for A, B and C Plants

Plant group	Amplicon name	Primers pair (see Table S1 for sequences)	Thermal cycling profile	Polymerase	Amplicon length (bp)
A	LBshort	NPTIIRBFOR<br>VBLBFOR	94°C, 5' (94°C, 30''/64°C, 40''/72°C, 45'') x 30 cycles, 72°C, 5'	Taq	1052
	VB1	VB1FOR<br>VB1REV	94°C, 5' (94°C, 30''/66°C, 30''/72°C, 30'') x 30 cycles, 72°C, 5';	Taq	257
	VB2	VB2FOR<br>VB2REV	94°C, 5' (94°C, 30''/66°C, 30''/72°C, 30'') x 30 cycles, 72°C, 5';	Taq	280
	RBshort	UBILBREV<br>VBRBREV	94°C, 5' (94°C, 30''/65°C, 45''/72°C, 1') x 30 cycles 72°C, 5';	Taq	645
	LBext	NPTIIRBFOR<br>VB1FOR	94°C, 5' (94°C, 30''/64°C, 30''/72°C, 2,5') x 30 cycles, 72°C, 8';	Taq	2429
	VBext	VB2FOR<br>VB1REV	94°C, (2'/94°C, 15''/67°C, 30''/72°C, 2'15'') x 30 cycles, 72°C, 8';	Phusion	3050
	RBext	UBILBREV<br>VB2REV	94°C, 2' (94°C, 10''/67°C, 20''/72°C, 1') x 30 cycles, 72°C, 8';	Phusion	2526
B	LBshort	UBILBREV<br>VBLBFOR	94°C, 5'(94°C, 30''/64°C, 40''/72°C, 45'') x 30 cycles, 72°C, 5';	Taq	645
	VB1	VB1FOR<br>VB1REV	94°C, 5'(94°C, 30''/66°C, 30''/72°C, 30'') x 30 cycles, 72°C, 5';	Taq	257
	VB2	VB2FOR<br>VB2REV	94°C, 5' (94°C, 30''/66°C, 30''/72°C, 30'') x 30 cycles, 72°C, 5';	Taq	280
	RBshort (pPZP-*hemL*)	GR6SCFOR<br>VBRBREV	94°C, 5' (94°C, 30''/65°C, 45''/72°C, 1') x 30 cycles, 72°C, 5'	Taq	884
	RBshort (pPZP-*nptII*)	VBRBREV<br>NPTIIRBFOR	94°C, 5' (94°C, 30''/65°C, 45''/72°C, 1') x 30 cycles, 72°C, 5'	Taq	920
	LBext	UBILBREV<br>VB1FOR	94°C, 5' (94°C, 30''/64°C, 30''/72°C, 2'30'') x 30 cycles, 72°C, 8';	Taq	2022
	VBext	VB2FOR<br>VB1REV	94°C, 2' (94°C, 15''/67°C, 30''/72°C, 2'15'') x 30 cycles, 72°C, 8';	Phusion	3050
	RBext (pPZP-*hemL*)	GR6SCFOR<br>VB2REV	94°C, 2' (94°C, 10''/67°C, 20''/72°C, 1') x 30 cycles, 72°C, 8';	Phusion	2764
	RBext (pPZP-*nptII*)	NPTIIRBFOR<br>VB2REV	94°C, 2' (94°C, 10''/67°C, 20''/72°C, 1') x 30 cycles, 72°C, 8'	Phusion	2800

Table S2 - (continue)

Plant group	Amplicon name	Primers pair (see Table S1 for sequences)	Thermal cycling profile	Polymerase	Amplicon length (bp)
C	LBshort	UBILBREV<br>VBLBFOR	94°C, 5'(94°C, 30''/64°C, 40''/72°C, 45'') x 30 cycles, 72°C, 5';	Taq	645
	VB1	VB1FOR<br>VB1REV	94°C, 5'(94°C, 30''/66°C, 30''/72°C, 30'') x 30 cycles, 72°C, 5';	Taq	257
	VB2	VB2FOR<br>VB2REV	94°C, 5' (94°C, 30''/66°C, 30''/72°C, 30'') x 30 cycles, 72°C, 5';	Taq	280
	RBshort	VBRBREV<br>NPTIIRBFOR	94°C, 5' (94°C, 30''/65°C, 45''/72°C, 1') x 30 cycles, 72°C, 5'	Taq	920
	LBext	UBILBREV<br>VB1FOR	94°C, 5' (94°C, 30''/64°C, 30''/72°C, 2'30'') x 30 cycles, 72°C, 8';	Taq	2022
	VBext	VB2FOR<br>VB1REV	94°C, 2' (94°C, 15''/67°C, 30''/72°C, 2'15'') x 30 cycles, 72°C, 8';	Phusion	3050
	RBext	NPTIIRBFOR<br>VB2REV	94°C, 2' (94°C, 10''/67°C, 20''/72°C, 1') x 30 cycles, 72°C, 8'	Phusion	2800

Table S3. List of the primers used in this work

Primer name	Sequence 5'-3'
LBnA1	cacaattccacacaacatacgagccggaag
LBnA2	cagtcgggaaacctgtcgtg
LBnA3	cgtccgcaatgtgttattaagttgtctaagcgtc
LBn1	gtttttgatgtatgtgacaaccctcgggattgttg
LBn2	gtgctatgtgtctgtcgagac
LBn3	cagtcgggaaacctgtcgtgccagc
RBn1	gattgaatcctgttgccggtcttgcgatg
RBn2	tgattagagtcccgcaattatac
RBn3	ccttgcagcacatccccctttcgcc
GR6SCFOR	gcagtttgaggcgggcttta
NPTIIRBFOR	catagcgttggctacccgtga
VBLBFOR	catgctaccctccgcgagat
VB1REV	cttcagcagagcgcagatacca
VB1FOR	tcagttcggtgtaggtcgttcg
VB2FOR	gcccattcttgagtcccgtatc
VB2REV	gaaagttgacccgcttcatgg
VBRBREV	gaagacggctgcactgaacg
RBINTFOR	cagttttctctttttgtcgaacg
RBINTREV	ctatattatactcaacccaatgagc
UBILBREV	cagtctttatgctcattgggttga
NPTIIshFOR	gcgataccgtaaagcacgag
NPTIIshREV	agcacgtactcggatggaag
PICA FOR	tatgacgagagccgcaacca
PICA REV	gacatgcacgatgccggtta
